# Utilizing Three-Dimensional Head-Lesser Trochanter Distance Could Further Reduce Leg Length Inequality in Primary Bipolar Hemiarthroplasty

**DOI:** 10.3390/jcm11216303

**Published:** 2022-10-26

**Authors:** Seungbae Oh, Yong-Sik Kim, Soon-Yong Kwon, Young-Wook Lim, Hyunwoo Park, Jongwoo Park, Joo-Hyoun Song

**Affiliations:** 1 Department of Orthopedic Surgery, St. Vincent’s Hospital, College of Medicine, The Catholic University of Korea, Seoul 06591, Korea; 2 Department of Orthopedic Surgery, Seoul St. Mary’s Hospital, College of Medicine, The Catholic University of Korea, Seoul 06591, Korea

**Keywords:** three-dimensional head-lesser trochanter distance, hemiarthroplasty, leg length inequality

## Abstract

Background: The aim of this study was to investigate whether the use of three-dimensional (3-D) computed tomography (CT)-based head-lesser trochanter distance (HLD) could reduce leg length discrepancy (LLD) more than the use of a two-dimensional (2-D) plain film method in primary bipolar hemiarthroplasty. Methods: Propensity score matching (PSM) analysis was used to adjust the confounding factors. A retrospective comparative analysis of 128 patients was performed. In the control group, the leg length was equalized using the 2-D, plain film-based HLD. In the study group, primary bipolar hemiarthroplasty was performed using the 3-D CT-based HLD method. Postoperative LLDs were compared between the two groups using the method of Ranawat. In addition, the Harris hip score (HHS) was evaluated and compared at one year after surgery. Results: A significant difference was observed in mean postoperative LLD between the 2-D HLD group and the 3-D CT HLD group: 1.6 ± 1.2 mm (range, 0.1–6.0 mm) and 1.1 ± 1.2 mm (range, 0.1–5.1 mm), respectively (*p* < 0.05). Additionally, a higher percentage of patients in the 3-D CT HLD group had an LLD of less than 2 mm. The mean HHS at one year after surgery showed no significant difference between the two groups. Conclusions: To minimize the occurrence of LLD, HLD measurement from a CT scanner may be more accurate than an X-ray. The 2-D and 3-D HLD differences in the 3-D CT HLD group were statistically significant. Using a 3-D, CT-based HLD method might decrease the possibility of an LLD over 2 mm.

## 1. Introduction

Approximately 1.7 million hip fractures occurred worldwide in 1990 [1]. It has been estimated that the incidence of hip fracture will rise to be between 4.5 million and 6.3 million worldwide by year 2050 [2,3]. Femoral neck fracture and intertrochanteric fracture represent the majority of hip fractures [4].

Hip arthroplasty might be one of solutions for femoral neck fractures. However, during surgery, leg length discrepancy (LLD) is one of the most challenging complications, even for highly skilled surgeons. It is associated with sciatic nerve palsies, lower back pain, abnormal gait, and dislocation [5,6,7,8,9,10,11].

Previously, many methods have been described for decreasing LLD. Preoperative templating or many intraoperative techniques can be used. In recent years, computer navigation systems and robotic surgeries have become important in hip arthroplasty. However, the extent to which navigation systems and robotic-assisted hip arthroplasty contribute to leg length inconsistency and the degree of additional accuracy remain controversial, and the results are inconsistent [12,13,14,15].

A method using heathy side head-lesser trochanter distance (HLD) and a picture archiving communication system (PACS) has also been reported [16,17,18]. The uninvolved HLD can serve as a reference for controlling HLD on the affected side [19].

**Figure 1:**
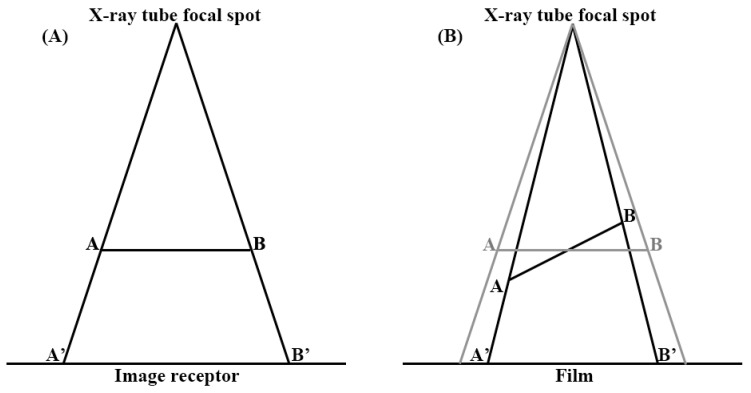
Magnification and distortion of the plane film. Objects further away from the focal spot are magnified, compared to those that are closer (**A**). Size and shape of image vary, depending on the amount of tilting (**B**).

However, plane radiographs are two-dimensional (2-D) representations of three-dimensional (3-D) objects. Superposition can lead to the loss of all depth information and ambiguity for the relative sizes of objects at different depths. Furthermore, it directly overlays objects, in such a way that it can become difficult or impossible to distinguish one from the other or to identify some objects. The object is also tilted, with respect to the plane of the film. The size and shape of image can also vary with the amount of tilting and angulation. Magnification and distortion can appear when using flat radiography (Figure 1).

Some trials have been conducted to correct errors due to depth difference. Attaching a real sized 10 cm reference bar can help minimize the error. To correct for magnification, some methods have been proposed [18,20]. However, it is hard to correct for the distortion of a plain film because angles of objects are various. Utilizing a computed tomography (CT) scanner is one way to reduce magnification and distortion at the same time. After measuring a 3-D HLD using a CT scanner, this measurement can be used intraoperatively to reduce LLD in hip arthroplasty.

The purpose of this study was to determine if using 3-D HLD could decrease leg length discrepancies in primary bipolar hemiarthroplasty. The hypothesis of this study was that utilizing 3-D HLD in CT could significantly decrease LLD in patients undergoing primary bipolar hemiarthroplasty.

## 2. Materials and Methods

### 2.1. Study Design and Patients Group

This study was reviewed and approved by the institutional review boards (IRBs) of the authors’ affiliated institutions.

A comparative analysis of patients treated using two-dimensional (2-D) head-lesser trochanter distance (HLD) and three-dimensional (3-D) HLD between January 2016 and December 2020 was performed. Propensity score matching (PSM) analysis was performed to adjust for confounding factors and reach similar baseline characteristics.

Inclusion criteria were: (1) hip bipolar hemiarthroplasty performed for displaced intracapsular femoral neck fractures using modified Gibson’s posterolateral approach between January 2016 and December 2020; (2) those who did not complain of leg length discrepancy (LLD) before fracture; (3) radiological and clinical data were available preoperatively and immediate postoperatively, as well as two weeks and one year after surgery; (4) subjects who used standard Corail femoral stems with a caput-collum-diaphyseal (CCD) angle (135 degree).

Exclusion criteria were: (1) subjects with fracture extending to the lesser trochanter; (2) those who complained of LLD before surgery; (3) subjects with a history of previous surgery on the contralateral hip; (4) those with pathologic fractures; (5) those with a follow-up loss; and (6) subjects who used the femoral stem with different CCD angles.

**Figure 2:**
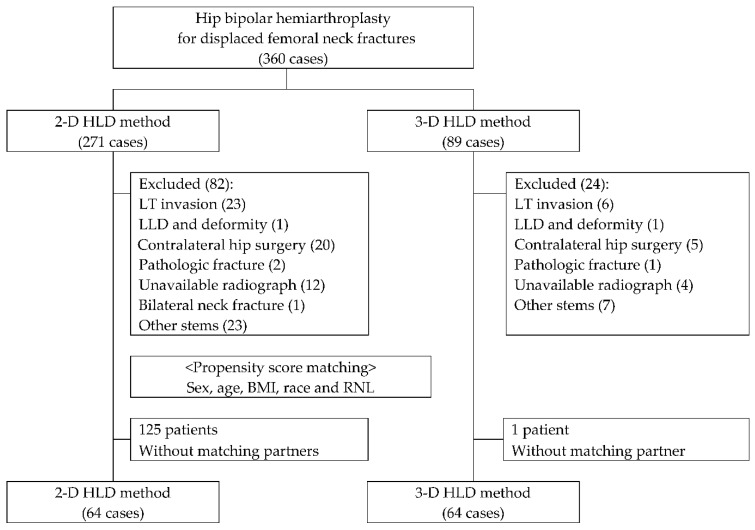
Classification of patients. HLD: head-lesser trochanter distance, LT: lesser trochanter, LLD: limb length discrepancy, BMI: body mass index, RNL: relative neck length.

Between January 2016 and December 2020, a total of 360 consecutive patients who were admitted to our institute underwent primary bipolar hemiarthroplasty. Among them, 106 patients were excluded, due to: fractures extending to lesser trochanter (29 patients), complaint of LLD before admission (2 patients), prior contralateral hip surgery and deformity (25 patients), pathologic fractures (3 patients), no appropriate radiograph for analysis (16 patients), simultaneous traumatic fractures of both femoral neck (1 patient), or undergoing surgery using a stem with different CCD angles (30 patients) (Figure 2).

**Figure 3:**
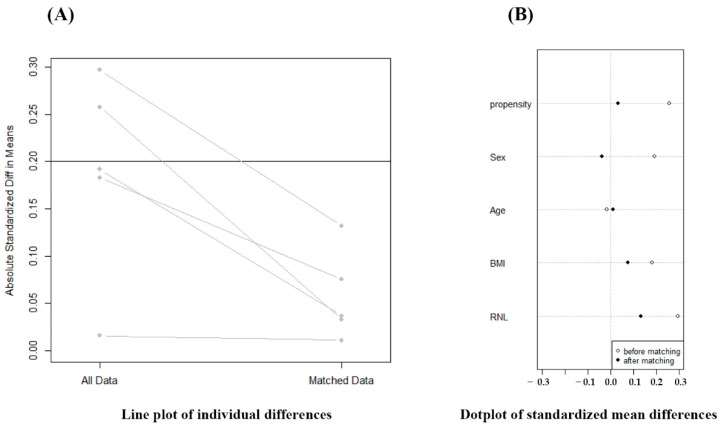
Evaluation of outcome of propensity score matching (PSM). PSM analysis was performed to adjust the confounding factors to reach similar baseline characteristics.

Among the remaining 254 patients, 65 patients were treated utilizing 3-D HLD in the later phase of study. These patients were matched to the patients utilizing 2-D HLD in the plane film in the early phase of the study. HLD was known to be affected by sex, age, BMI, race, and relative neck length (RNL) [21]. Thus, matching criteria were as follows: sex, age, BMI, and RNL, since study subjects were all Koreans. Line plot of individual differences and dot plot of standardized mean differences were shown to examine the outcome of PSM (Figure 3). Finally, a total of 128 patients who had radiographs for review and follow-up were enrolled.

### 2.2. Radiologic Parameters

The study was conducted under the assumption that the leg length would be the same if the actual distance from the center of the femoral head to the superior end of the lesser trochanter was about the same for both hips. For all surgeries, preoperative plan and templating were performed on standardized plain radiographs with a PACS. Radiographs from an AP view of the pelvis centered over the pubic symphysis with the healthy side hip in 10⁰ to 15⁰ of internal rotation were used. Pelvic tilt was judged by looking at the pubic symphysis to the sacrococcygeal distance, with normal values of 32 mm (range 8–50 mm) in females and 47 mm (range 15–72 mm) in males [22].

**Figure 4:**
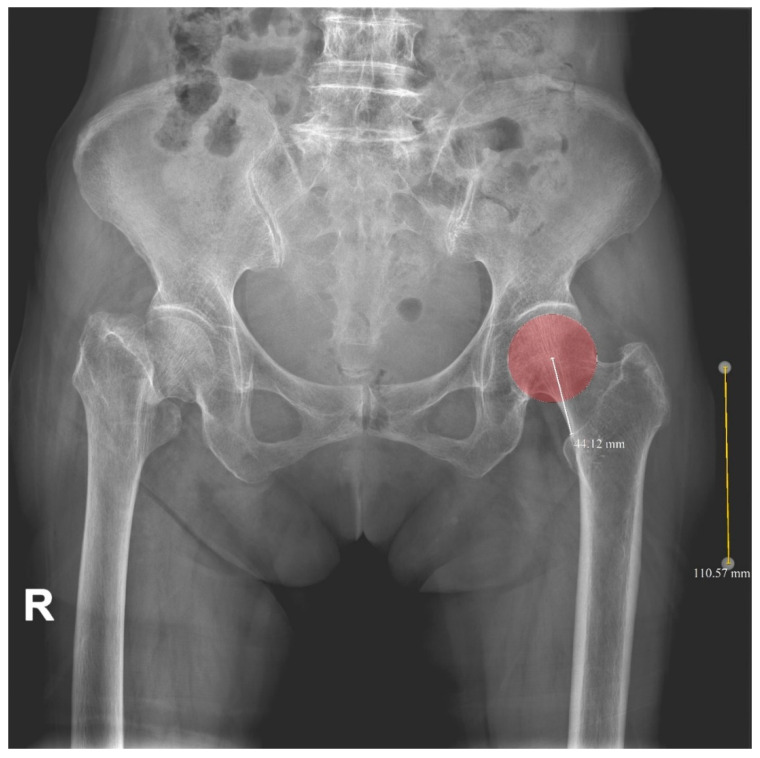
Two-dimensional head-lesser trochanter distance (2-D HLD). Magnification was corrected through a 10-cm sized magnification marker. R: right-sided on radiographs.

**Figure 5:**
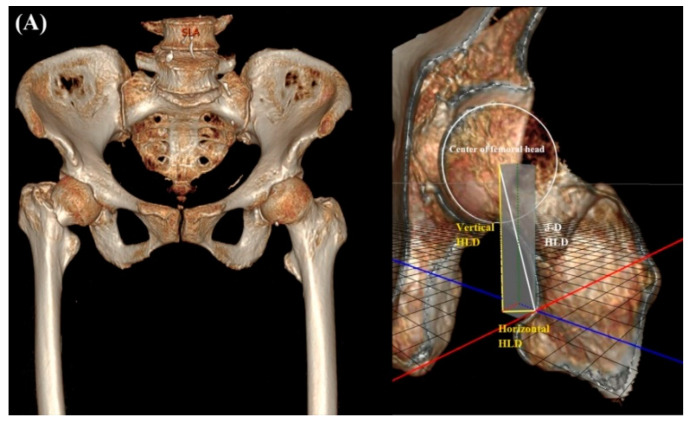

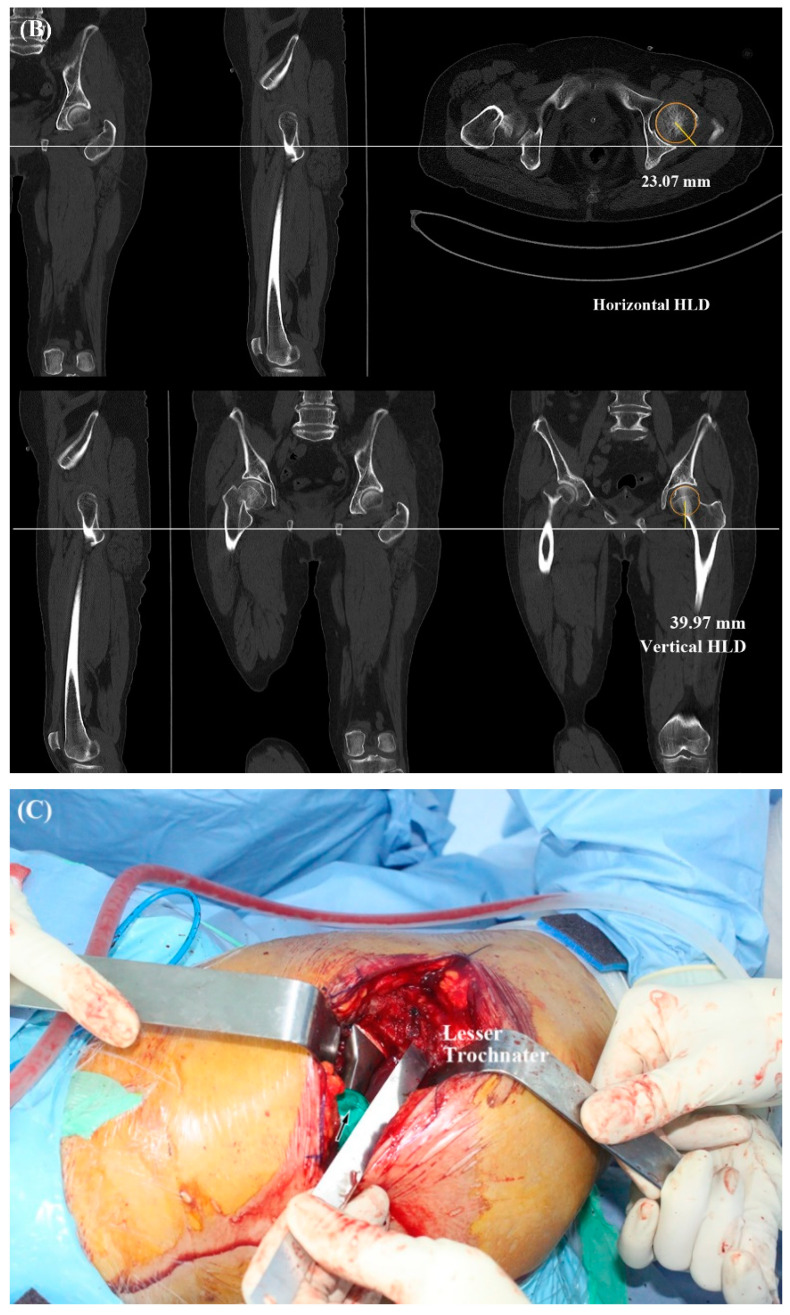
Three-dimensional head-lesser trochanter distance (3-D HLD) was calculated using the Pythagorean theorem with the horizontal HLD and vertical HLD as variables (**A**,**B**). Adjust the modular neck system to match 2-D or 3-D HLD measurements (**C**).

In the early phase of this study, 2-D HLD from plain radiographs was used to minimize the LLD. The HLD of the opposite hip was measured on a PACS plain radiograph preoperatively. After magnification was corrected through a 10-cm sized magnification marker, the HLD was equalized by selecting a suitable modular head during the surgery (2-D HLD method) (Figure 4). In the later phase, 3-D HLD of the opposite hip was measured from CT scan. On the CT scan, distance from the center of the femoral head to the superior end of the lesser trochanter was calculated in three-dimension. Horizontal HLD was measured in the axial cut of CT. Vertical HLD was measured in the coronary cut of CT. The horizontal HLD reflects both *x*-axis and *y*-axis of the HLD. The vertical HLD reflects the *z*-axis of the HLD. Three-dimensional HLD was calculated using the Pythagorean theorem, with horizontal HLD and vertical HLD as variables (3-D HLD method) (Figure 5A,B).

**Figure 6:**
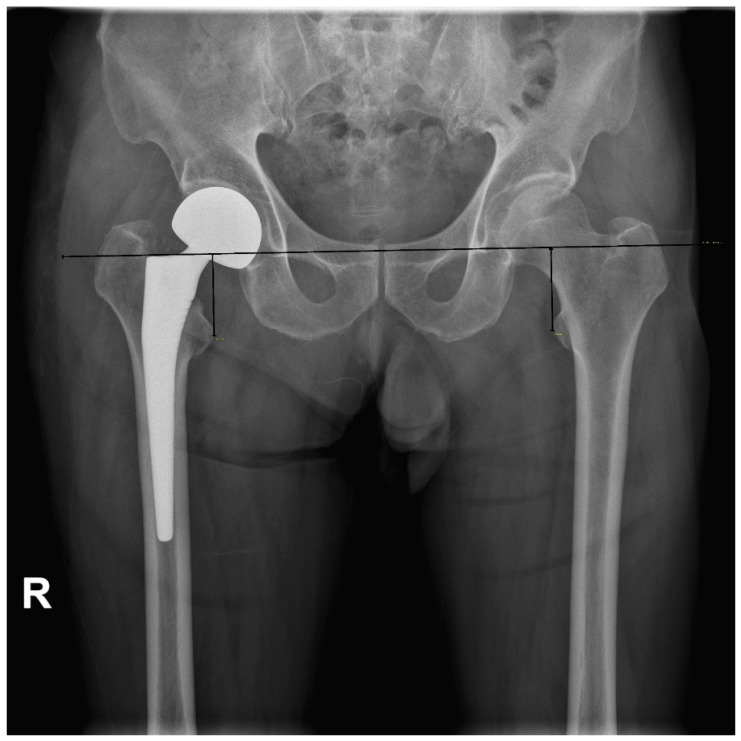
Leg length discrepancy measurement postoperatively, described by Ranawat et al. [23].

The LLD immediate was assessed postoperatively using the method described by Ranawat et al. [23]. Standard AP radiograph of the pelvis with similar size of the lesser trochanter and the degree of overlap of the medial cortex of the greater trochanter on both sides was obtained with the patient in a supine position, centered on the pubic symphysis for both hips, without pelvic tilt or rotation. On an AP radiograph of the pelvis, a horizontal line was drawn through the inferior aspect of teardrops (the perpendicular distance between the reference line and lesser trochanter) (Figure 6). Two investigators independently evaluated results with each method to assess interobserver variation and reliability. One investigator in each group repeated measurements one week later to assess intraobserver variability for the corresponding technique.

### 2.3. Operative Technique

All surgeries were performed by a single attending surgeon. Surgical procedures were generally the same. Patients were placed in a lateral decubitus position. The hip was exposed by the posterolateral approach and broached serially in 1 size increments, until a press-fit was achieved. The rasp was inserted to the proximal femur to the same degree as the neck. Additionally, to prevent varus or valgus orientation, it was checked using C-arm during surgery. After the surgeon positioned the femoral component, based on preoperative templating or evaluation with a CT scanner, a modular head with a proper neck length was introduced. The HLD was equalized by selecting a suitable modular head during surgery, with 2-D or 3-D HLD methods. Final appropriate trial head was measured intraoperatively for restoration of head-lesser trochanter distance (Figure 5C). A 28 mm inner head was used with a neck length from 1.5 mm (short) to 8.5 mm (long). Appropriately sized bipolar shell, as measured, was then assembled.

The neck shaft angle of the Corail stem (DePuy, France, SAS) was constant at 135 degrees. Since only standard offsets were used, there was no variance, depending on the type of stem.

### 2.4. Statistical Analysis

Pearson’s chi-square test was used to compare differences of categorical variables. Independent samples t-test was used to compare continuous variables.

Sample size was estimated by utilizing an effect size of 0.5, an acceptable alpha error of 0.05, and a beta error of 0.2 to ensure power of 80%. Calculations indicated that it would be necessary to include at least 64 patients to compare means between the two groups using independent sample *t*-tests.

The Kolmogorov–Smirnov and Shapiro–Wilk tests were used to assess normality of distribution. Reproducibility was assessed based on the intraclass correlation coefficient (ICC). Two examiners independently measured RNL, 2-D HLD, 3-D HLD, LLD, canal flare index (CFI), cortical index ratio (CI), and Dorr type. Intra-observer reliability was assessed using the values measured by each examiner. Inter-rater reliability was also measured by comparing the means of two examiners. Reliability measurements were reviewed, and the results reached substantial to almost perfect agreement. All statistical analyses were performed using SPSS 25 software (SPSS, Inc., Chicago, IL, USA).

## 3. Results

**Figure 7:**
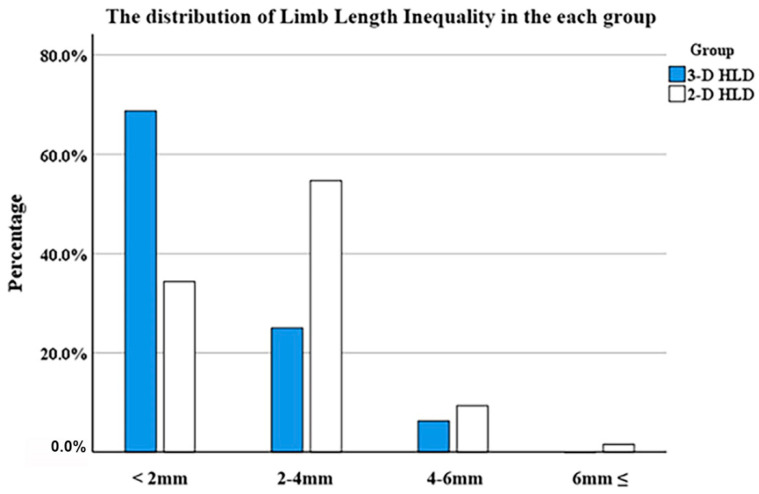
Leg length discrepancy distribution in the 2-D and 3-D HLD groups.

In both groups, basic patient demographics did not significantly differ, with the exception of the follow-up period (Table 1). There was a significant difference in mean postoperative limb length discrepancy (LLD) between the plain X-ray head-lesser trochanter distance (HLD) group and the three-dimensional (3-D) computed tomography (CT) HLD group: 1.6 ± 1.2 mm (range, 0.1–6.0 mm) and 1.1 ± 1.2 mm (range, 0.1–5.1 mm), respectively (*p* < 0.05). Additionally, a higher percentage of patients in the 3-D CT HLD group had an LLD of less than 2 mm than in the plain X-ray HLD group (*p* < 0.05) (Table 2, Figure 7). The 2-D and 3-D HLD difference in the 3-D CT HLD group was 2.7 ± 2.0 mm, which was statistically significant (*p* < 0.05) (Table 2).

Mean Harris hip score (HHS) at one year after bipolar hemiarthroplasty was 86.9 (range, 65–93) in the 2-D plain X-ray HLD group and 86.2 (range, 63–98) in the 3-D CT HLD group, showing no significant difference between the two groups.

## 4. Discussion

To restore the contralateral normal hip head-lesser trochanter distance (HLD), measurement from a computed tomography (CT) scanner might be more accurate than from an X-ray. Horizontal HLD reflects both the *x*-axis and *y*-axis of the HLD. Vertical HLD reflects the *z*-axis of the HLD. The difference between 3-D HLD and 2-D plain X-ray HLD did not seem to be very large numerically. However, the difference was statistically significant. In practice, such a difference in length might have some effects on lower limb inequality. Magnification and distortion can be drastically reduced with 3-D HLD, rather than with 2-D HLD, and the actual length can be accurately reflected, leading to good results.

Due to the retrospective study design, the two groups might have different backgrounds. Propensity score matching (PSM) analysis was recommended to adjust for confounding factors to reach comparable baseline characteristics. A dot plot of the standardized mean differences and a linear plot of the individual differences were obtained to examine the outcome of PSM.

Most subsidence occurs within one year after the surgery [24]. Therefore, it would be appropriate to evaluate LLD by surgical method immediately postoperatively. However, by measuring the Harris hip score (HHS) one year later, the pain and functional aspects during follow-up were not neglected.

The mean postoperative LLD in the 3-D HLD group was 1.1 mm. This result was largely due to the modular neck system. During surgery, the HLD was equalized by selecting a suitable modular neck, and the difference between necks was 3.5 mm. Another reason was that error might occur when measuring HLD during surgery because of the ambiguity of the superior border of lesser trochanter. To reduce such error, several measurements were made in the process of identifying HLD during surgery. Furthermore, for the CT scan from the hospital, the film cut was usually 1.5–2 mm in the axial, coronary, and sagittal planes, except for special cases. Therefore, there are clearly technical limitations in the process of matching HLD equally.

Limb length inequality was found in most people in the general population [25]. Additionally, it is known that right and left hips differ by 2.0–2.5 mm in their offset [19,26]. For these reasons, matching the opposite leg length is not always right. One study has reported how to determine target limb length and offset in total hip arthroplasty, focusing on disease severity and patient perception [27]. Previous studies have shown that achieving limb length and offset within 5 mm can result in acceptable outcomes [28,29,30].

Recently, various attempts have been made into four categories using intraoperative measurements, fluoroscopic guidance, navigation, or robot-assisted surgery to reduce limb length inequality, with varying results obtained by many authors (Table 3). Gonzalez et al. and Matsuda et al. reported how actual HLD can be measured before surgery and reproduced at the surgery site using a modular neck system with an average postoperative LLD between 1.71 mm and 2 mm [16,17]. Lim et al. reported better results by correcting for magnification [18]. In the present study, efforts were made to further reduce lower limb length inequality by correcting for magnification and distortion altogether.

Konyves et al. have found that femoral stem positioning is associated with 98% of limb length inequality during total hip arthroplasty [47]. These results indicate that acetabular component positioning has minimal contribution to the LLD after total hip arthroplasty (THA). Thus, the present study focused on LLD’s contribution of the femoral component by identifying the HLD of both femurs in bipolar hemiarthroplasty. Randomized controlled trial and more studies for hemiarthroplasty (HA) and THA using 3-D HLD are needed, and this requires further investigation.

This study has some limitations. Since many recent studies, including this investigation, are studies to reduce the leg length inequality of about a few millimeters or less, in the end, it may ultimately raise questions about clinical relevance. It is known that changes in limb length or offset within 5 mm not only cause no symptoms, but also do not cause significant changes in functional scores, such as HHS or Oxford hip score (OHS) [28,29,30,47]. In this respect, this study can find its meaning in that it might reduce LLD, compared to the previous studies using HLD, but it has limitations in that the clinical relevance and effectiveness may be lowered.

In addition, it is necessary to point out the cost effectiveness of the CT scan performed in the experimental group. CT scans are expensive, labor intensive, and require additional radiation exposure to the patient. Additionally, CT capacity and immediate availability, which should be performed in patients with femoral neck fractures who require urgent operation, should be pointed out.

Although there was an unavoidable limitation of additional radiation exposure, efforts were made to increase availability by performing CT immediately after plane X-ray when a patient with a femoral neck fracture visits the emergency room. The study was conducted while reducing medical expenses that could be a burden to patients by compensating for the cost of CT, to some extent, through the National Medical Insurance.

Although we tried to control the confounding factors of the experimental and control the groups through matching, the retrospective study design could not give the robustness of data offered by a prospective data collection and could not completely eliminate the selection bias. A total of 29.4% of geriatric patients were excluded because of reasons including incomplete follow-up, and this situation would inevitably cause a selection bias.

Additionally, with the focus on LLD, instability caused by stem version or varus orientation, and offset might have been overlooked. Basically, standard Corail stems with 135° neck-shaft angle were used equally in this study. Femoral offset and global offset are also important considerations. Mahmood et al. found that a reduction in global femoral offset after THA was associated with a loss of abductor muscle strength [48]. Additionally, failure to correctly consider these parameters can result in joint instability and dislocation, especially in patients with a stiff spine [49].

Radiographic LLD measurements made on the plain X-ray are also susceptible to error, since the horizontal dimensional parameters are influenced by variations in the positioning of the pelvis and proximal femurs, as well as the divergence of the X-ray beams [50]. We tried to reduce errors by obtaining a radiograph centered on the pelvis without pelvic tilt or rotation.

In the future, more investigations are needed to reduce selection bias through prospective studies. Prospective studies are also needed to compare total hip arthroplasty and bipolar hemiarthroplasty. These studies can help us know the usefulness of the 3-D HLD method, including the attributions of the acetabular and femoral components for LLD.

## 5. Conclusions

To minimize the occurrence of LLD, HLD measurement from a CT scanner may be more accurate than an X-ray. The 2-D and 3-D HLD difference in the 3-D CT HLD group was statistically significant. Using the 3-D CT-based HLD method might decrease the possibility of an LLD over 2 mm.

## Figures and Tables

**Table 1:** Patients’ demographics.

Variable	Plane X-ray HLD Group	3-D CT HLD Group	*p* Value
Number of patients (hips)	64	64	
Side (right/left)	33/31	36/28	0.595
Male/female	14/50	15/49	0.833
Age	79.3 ± 8.7	79.3 ± 8.4	0.951
Body mass index (BMI)	21.9 ± 3.2	22.2 ± 3.4	0.633
Relative neck length (RNL)	−0.3 ± 0.7	−0.2 ± 0.6	0.494
Follow-up period	19.5 (12–61)	14.2 (12–24)	0.001
Prosthesis	Corail 64	Corail 64	1.000
2-D HLD	43.4 ± 4.5	43.1 ± 3.9	0.710
3-D HLD	-	45.9 ± 3.6	N/A
Prosthesis			
Corail 8	0	2	
Corail 9	2	1	
Corail 10	3	5	
Corail 11	8	10	
Corail 12	17	13	
Corail 13	17	17	
Corail 14	8	11	
Corail 15	7	4	
Corail 16	2	1	0.327
Neck length			
Short neck	48	49	
Standard neck	13	9	
Long neck	3	6	
Extra-long neck	0	0	0.767
Canal flare index (CFI)	3.12 ± 0.57	2.95 ± 0.56	0.085
Cortical index ratio (CI)	0.48 ± 0.07	0.47 ± 0.07	0.229
Dorr type A	5	3	
Dorr type B	25	24	
Dorr type C	34	37	0.724

CT: computed tomography, HLD: head-lesser trochanter distance.

**Table 2:** Patients’ postoperative data.

Parameter	Plane X-ray HLD Group	3-D CT HLD Group	*p* Value
Mean LLD	1.6 ± 1.2	1.1 ± 1.2	0.024
<2 mm	22 (34.4%)	44 (68.8%)	
2–4 mm	37 (57.8%)	16 (25.0%)	
4–6 mm	4 (6.3%)	4 (6.3%)	
≥6 mm	1 (1.6%)	0 (0.0%)	0.001
Harris hip score	86.9 (65–93)	86.2 (63–98)	0.507

CT: computed tomography, HLD: head-lesser trochanter distance, LLD: limb length discrepancy

**Table 3:** Review of the literature reported by many authors with various results with intraoperative methods.

Study	Mean LLD (mm)	Methods
McGee and Scott (1985) [31]	No results presented	A Steinmann pin was driven in 2 cm superior to the acetabulum and bent into a “u” shape; a mark was made at the point where the free end of the “u” contacted the greater trochanter
Woolson (1990) [32]	2.8	Comparing the dimensions of the resected bone with the dimensions replaced by the prosthesis
Jasty et al. (1996) [33]	5.4	Use of mechanical jigs and measuring calipers
Bose (2000) [34]	3.4	Use of measuring calipers (the Acculength hip gauge device)
Ranawat et al. (2001) [23]	7.4	A vertical Steinmann pin at the infracotyloid groove of the acetabulum
Shiramizu et al. (2004) [35]	2.1	Use of measuring L-shaped caliper
Gonzalez et al. (2005) [16]	1.71	Measuring between the proximal edge of the lesser trochanter and the center of rotation of the femoral head (HLD)
Matsuda (2006) [17]	2.0	Measuring the actual HLD preoperatively and reproducing it in the operative field with a modular neck system
Ecker et al. (2007) [36]	1.3	Computed tomography-based navigation
Mainard (2008) [37]	4.4	Imageless navigation
Lim et al. (2013) [18]	1.5	Measuring head to lesser trochanter length using PACS and reproducing it in the operative field with a modular neck system
Brown et al. (2014) [38]	5.2	Imageless navigation
Ogawa et al. (2014) [39]	2.9	Measuring with PCA limb lengthening gauge
Tsai et al. (2016) [40]	0.7	Robot-assisted technology
Bingham et al. (2018) [41]	1.1	Fluoroscopic guidance
Wang et al. (2019) [42]	4.4	Measuring ratio of contralateral femoral head and the distance of HLD using PACS
Clement et al. (2021) [43]	2.3	Robot-assisted technology
Herrero et al. (2021) [44]	1.02	Fluoroscopy-based navigation
Chen et al. (2022) [45]	2.5	Use of measuring horizontal calibrator
Stewart et al. (2022) [46]	3.79	Fluoroscopic guidance

LLD: limb length discrepancy, PCA: proper noun, limb lengthening gauge (Stryker, Mahwah, NJ, USA), HLD: head-lesser trochanter distance, PACS: picture archiving communication system.

## Data Availability

The data collected for this study, including individual patient data, will not be made available.
